# Adipose-Derived Molecules–Untouched Horizons in Alzheimer’s Disease Biology

**DOI:** 10.3389/fnagi.2020.00017

**Published:** 2020-02-13

**Authors:** P. B. Tirupathi Pichiah, Devaraj Sankarganesh, Sankarganesh Arunachalam, Shanmugam Achiraman

**Affiliations:** ^1^Department of Environmental Biotechnology, Bharathidasan University, Tiruchirappalli, India; ^2^Department of Biotechnology, School of Bio and Chemical Engineering, Kalasalingam Academy of Research and Education, Krishnankoil, India; ^3^Department of Microbial Biotechnology, Bharathiar University, Coimbatore, India

**Keywords:** adipose tissue, obesity, adipose-derived molecules, Alzheimer’s disease, autophagy

## Abstract

The global incidence of Alzheimer’s disease (AD) is on the rise with the increase in obesity and metabolic disease epidemic. Obesity is co-morbid with the increase in mass of adipose tissue, which secretes numerous molecules that are biologically important. Obesity and its associated conditions are perhaps involved in the causative pathway of AD. Immunologically important cytokines such as IL-1β, IL-10, and IL-18, which are released by adipose tissue, are also found to be associated with AD. Besides, the expression of IL-6, IFNγ, and TNF alpha are also associated with AD. Ang-I and Ang-II are found to mediate the progression of AD. Complement factors B, C4b, and H are differentially expressed in AD. Overall, several adipocyte-derived cytokines are found to be dysregulated in AD, and their role in AD remains to be studied. The induction of autophagy is a very promising strategy in the treatment of AD. A variety of adipose-derived molecules have been shown to modulate autophagy. However, very little literature is available on the role of adipose-derived molecules in inducing autophagy in microglial cells of AD. Understanding the role of adipose-derived molecules in the development of AD, especially in the induction of autophagy, would open up new avenues in devising strategies for the treatment of AD.

## Introduction

Alzheimer’s disease (AD) is one of the most prevalent neurodegenerative disorders. Individuals affected with AD show a decline in cognition and loss of self-dependency further leading to hospitalization and premature death. According to a recent WHO report, 60–70% of cases of dementia are contributed by AD. On the whole, it has been estimated that 5.2 million Americans of all ages have AD. Among this population, 5 million people are at the age of 65 or older (Alzheimer’s Association, 2006). Further, one-third of people around the age of 85 have AD (32%) (Hebert et al., 2013). Several studies suggest that both overweight and obesity tend to increase the risk of AD (Gustafson et al., 2003; Kivipelto et al., 2005; Rosengren et al., 2005; Whitmer et al., 2005). Obesity, on the other hand, is a predisposing factor for vascular dysfunction (Zhang and Reisin, 2000). Furthermore, a strong association between dementia and adiposity has been stated by many researchers. Naderali et al. (2009) suggested that obesity-mediated defects in insulin and glucose signaling may evoke AD (Naderali et al., 2009). It has also been observed that obesity-mediated progression of AD is likely to be more prevalent in men compared to women (Elias et al., 2003).

The brain is the master regulator of all the organ systems and functions through either direct or indirect modes. The indirect mode of communication between the brain and the organ systems involves the exchange of bioactive peptides that can cross the blood–brain barrier (BBB). Many of these bioactive peptides include a variety of cytokines and chemokines that are released from different organs and signal the brain. One of the major organs that produce a wide array of bioactive peptides is adipose tissue, and it is, therefore, now recognized as an endocrine organ (Trayhurn and Beattie, 2001; Kershaw and Flier, 2004). Adipose-derived molecules are the peptides that are specifically produced by adipose tissues. These peptides include hormones, enzymes, complement factors, immunomodulators, etc. An increase in adipose tissue mass or adiposity disrupts the homeostasis of these adipose-derived molecules. Expression levels of a variety of these molecules are directly proportional to adipose tissue mass (Benoit et al., 2004). Examples include leptin, adiponectin, apelin, etc (Azuma et al., 2003; de Courten et al., 2004). Adipose-derived molecules play a crucial role in the progression of AD (Warren et al., 2012). Thus the occurrence of AD may be linked to the magnitude of adiposity (Luchsinger and Gustafson, 2009). This review highlights the role of adipose-derived molecules in implicating AD that connects both obesity and AD. Furthermore, the possible roles of the molecules in inducing autophagy are discussed.

## Cytokines

Adipose tissues [both white adipose tissue (WAT) and brown adipose tissue (BAT)] are well known to produce inflammatory cytokines, which rise with adiposity (Coppack, 2001; Fain, 2006). These cytokines play vital roles in the progression of AD (Rubio-Perez and Morillas-Ruiz, 2012).

### Interleukins

Interleukins (IL) act as messengers and assist in communication between cells within and across the tissues. They regulate cell growth, differentiation, motility, the stimulation of immune responses, etc. Adipose tissues produce a wide variety of interleukins (Kern et al., 2001) including IL-1β, IL-4, IL-6, IL-8, IL-10, and IL-18. These interleukins have been known to correlate with AD.

### Interleukin 1β (IL-1β)

Interleukin 1β is a cytokine involved in immune modulation. The hyperglycemic condition accelerates the synthesis of IL-1β in the adipose tissue of both humans and rodents via thioredoxin interacting protein (TXNIP) (Koenen et al., 2011). A comparison of the post-mortem brain of AD patients and the control group indicated an increased level of IL-1β in the AD patients, specifically at the frontal cortex and hippocampus. François et al. (2013) have demonstrated the relationship between autophagy and IL-1β. Autophagy induced IL-1β in microglia through degrading inflammasomes (François et al., 2013). These findings shed light on the involvement of IL-1β in AD.

### Interleukin 4 (IL-4)

Interleukin 4 is a cytokine that is secreted by the adipose tissue (Ouchi et al., 2011). As an anti-inflammatory cytokine, IL-4 protects the brain from inflammation-induced damage. In amyloid precursor protein (APP)-transgenic mice, IL-4 enhances the degradation of amyloid beta (Aβ), thereby preventing the Aβ-induced cell death (Ouchi et al., 2011). In contrast, Chakrabarty et al. (2012) observed mIL-4 in the hippocampus favored amyloid deposition *in vivo* (Chakrabarty et al., 2012). Both studies revealed the association of IL-4 with AD. Besides, IL-4 is a well-known inducer of autophagy in B cells (Xia et al., 2018), which may also induce autophagy in brain cells.

### Interleukin-10 (IL-10)

Interleukin-10 is also an anti-inflammatory cytokine that is produced by the adipose tissue. It is otherwise known as the human cytokine synthesis inhibitor factor (CSIF). IL-10 is mostly produced by visceral adipose tissue of obese subjects (Juge-Aubry et al., 2005). Human WAT explants also produce IL-10 when exposed to tumor necrosis factor alpha (TNF alpha) and lipopolysaccharide (LPS). In the microglial cell, IL-10 is capable of suppressing the monocyte chemoattractant protein-1 (MCP-1) production in concert with the exposure of Aβ peptide. Furthermore, it also modulates the immune process associated with AD development (Szczepanik et al., 2001). Despite this, there is no sufficient information available to conclude the exact mechanism of IL-10 in the development of AD. Conversely, the role of IL-10 in cardiac autophagy is established (Samanta and Dawn, 2016), though not in brain cells.

### Interluekin-18 (IL-18)

White adipose tissue is one of the major sources of IL-18 (Wood et al., 2005). Sutinen et al. (2012) demonstrated that a high level of IL-18 increases Beta-secretase (beta-site APP cleaving enzyme-1) (BACE-1) (APP-cleaving enzyme) together with the γ-secretase complex in the brain (Sutinen et al., 2012). It also raises the level of Fe65, which regulates glycogen synthase kinase-3β (GSK-3β) by binding with the C-terminus of APP. Culture medium, when treated with IL-18, showed increased levels of soluble APP-β, thus exemplifying the importance of IL-18 in APP-β production. The elevated level of IL-18 in the brain for a prolonged period leads to AD (Bossù et al., 2010), possibly through increased Aβ. But the involvement of IL-18 in inducing autophagy remains elusive.

### Tumor Necrosis Factor Alpha (TNF Alpha)

Adipose tissue produces TNF alpha, which plays key roles in the inflammatory pathway (Sewter et al., 1999; Hoareau et al., 2010). Many studies with rodent models demonstrated that overexpression of TNF alpha in adipose tissue promotes insulin resistance (Hotamisligil et al., 1993, 1995). TNF alpha acts as an initiator of inflammation in the brain (Feldmann and Maini, 2003) and regulates neuroinflammation. A post-mortem study localized TNF alpha within the amyloidogenic plaque of AD patients’ brain (Dickson, 1997). Later, TNF alpha was found to be increased in the cerebrospinal fluid (CSF) of AD patients (Tarkowski et al., 2003). Consequently, a clinical trial in AD patients using a TNF alpha inhibitor (Etanercept) showed that TNF alpha inhibition could be a promising approach to control AD (Tobinick et al., 2006). Furthermore, TNF alpha has been suggested to inhibit autophagy in microglia (Jin et al., 2018). This could be due to the induction of autophagy caused by the inhibition of TNF alpha.

### Macrophage Migration Inhibitory Factor (MIF)

Adipose tissue secretes MIF (Skurk et al., 2005), which is an inflammatory cytokine of innate immunity. MIF is co-localized with Aβ-protein, promoting inflammation around the plaque areas, and is thus able to form amyloid-like fibrils. This notion validates its importance in neuroinflammation and plaque development (Oyama et al., 2000; Lashuel et al., 2005). Further, MIF is markedly increased in AD patients, indicating its importance in AD pathogenesis. MIF favors AD pathogenesis by accelerating the production of other cytokines (Popp et al., 2009; Bacher et al., 2010). An *in vitro* study using an MIF inhibitor in the neuroblastoma cell line revealed amelioration of the neurotoxic effect of MIF and Aβ protein production. Similar results were also observed with murine BV2 microglial cells (Bacher et al., 2010). This evidence sheds light on the potential of MIF inhibitors in developing treatment strategies for AD. However, the role of MIF is not studied in autophagy.

### Leptin

The mature adipocytes secrete leptin that assists the brain in deciding the level of energy intake. Leptin thus regulates feeding behavior, energy expenditure, and other activities. Leptin transport across the BBB is facilitated by leptin receptors (Schulz et al., 2011), which are abundantly found in the hypothalamus, neocortex, cerebellum, and medulla. Moreover, leptin receptors are also found in cells that produce orexigenic and anorexigenic neuropeptides (Jequier, 2002). These neuropeptides are crucial to maintaining the energy intake. Leptin is now counted as a biomarker in predicting AD progression (Lieb et al., 2009). A recent investigation showed that leptin attenuates the hyper-phosphorylation of tau protein. Conversely, the hippocampal tissue and CSF of AD patients revealed a high level of leptin and leptin receptors (King et al., 2018). Leptin has been shown to induce autophagy in neuronal cells (Li et al., 2018), but this has not been demonstrated in relation to AD.

## Growth Factors

### Angiopoietins [Angiopoietin-1 (Ang-1) and Angiopoietin-2 (Ang-2)]

Angiopoietin-1 is involved in the remodeling of WAT during weight loss and gain; this process not only involves changes in adipocytes but is also associated with the distinct changes in the density of blood vessels and nerve fiber. Several adipokines are involved in this process. For instance, Tie proteins remodel the adipose tissue through vascular maturation (Dallabrida et al., 2003). Adipocyte-derived stem cells produce Ang-1 when supplemented with growth factors in the medium. The production of Ang-1 is time-dependent, and knockdown of Ang-1 negatively affects endothelial regenerative capabilities (Takahashi et al., 2013). This proves that angiogenesis is partially regulated by Ang-1. Therefore, understanding the role of Ang-1 in angiogenesis would help to delineate obesity and other related conditions. Furthermore, angiogenic activation of the brain cell endothelium favors the formation of β-amyloid, which is the hallmark of AD (Vagnucci and Li, 2003). The measurement of serum Ang-1 in AD patients and control revealed significantly increased Ang-1 in AD patients. Ang-1 level in serum is, therefore, suggested as a complementary technique together with mental status examination to diagnose AD (Schreitmüller et al., 2012). However, further studies are warranted to validate Ang-1 as a potential marker of AD, and its role in autophagy remains unstudied.

Ang-2 is also important in the vascularization of adipose tissue, which shows greater plasticity by expanding or reducing its size throughout the lifespan. Ang-2 regulates either vascular remodeling or regression, through positive or negative regulation. It is closely connected with obesity because leptin has a crucial role in inducing the expression of Ang-2, which is independent of other angiogenic factors [e.g., vascular endothelial cell growth factor (VEGF)] (Cohen et al., 2001). Tabata et al. (2009) reported the secretion of an angiopoietin-like protein-2 by adipocytes and considered it as a marker of adiposity (Tabata et al., 2009). Ang-2 promotes vascularization of subcutaneous WAT and improves metabolic homeostasis, and it is therefore has a promising role in mitigating high-fat diet-induced obesity (An et al., 2017). However, the function of Ang-2 is not yet established in AD and neither is autophagy.

### Fibroblast Growth Factors (FGFs)

Fibroblast growth factors consist of 22 members and are secreted both by WAT and BAT. The FGFs produced by WAT are involved in a variety of functions including metabolism, and neural development (Itoh and Ohta, 2014). FGF-1 is significantly increased in WAT during obesity than FGF-2 (Mejhert et al., 2010). FGF-9 regulates the expansion of BAT under cold-induced conditions (Shamsi et al., 2018). FGFs have a potential therapeutic application against obesity, and understanding their molecular mechanism is thus pivotal (Nies et al., 2016). FGFs, on the other hand, are required for the development of various brain areas. For instance, the supplementation of FGFs increases neuronal survival in the midbrain, hippocampus, etc (Matsuda et al., 1990). The occurrence of FGFs is mostly associated with the plaques, and this was evidenced by the focal immunoreactivity of neuritic plaques (Stopa et al., 1990). The systemic administration of FGF in APP-23 transgenic mice resulted in decreased Aβ formation and tau synthesis (Katsouri et al., 2015). Overall, it is interesting to explore FGFs for their beneficial role in mitigating AD. FGF-2 has been reported to regulate autophagy in non-small-cell lung cancer cells (Yuan et al., 2017), which leads to the speculation that it might induce autophagy in controlling AD.

### Hepatocyte Growth Factor (HGF)

Hepatocyte growth factor is expressed as an angiogenic factor in adipose tissue. HGF expression is reduced during adipocytes differentiation and upregulated during hypoxia (Chu et al., 2009). In the human brain, HGF is localized in the astrocytes of white matter (Tsuboi et al., 2003), and its expression level varies in response to brain injury (Yamada et al., 1994). Therefore, its concentration is useful to assess the extent of damage in the white matter of AD patients (Laterra et al., 1997). It remains to be further elucidated whether HGF could be used as a biomarker for AD. HGF and MET together induce autophagy in certain cancer cells (Huang, 2018). However, the role of HGF in controlling autophagy in brain cells is not established.

### Insulin-Like Growth Factor-1 (IGF-1)

Insulin-like growth factor-1 is responsible for the increase in tissue mass through the regulation of growth hormones. In adipose tissue, the differentiation of pre-adipocytes to adipocytes is favored by the growth factors. Particularly, the newly differentiated adipocytes are highly sensitive to IGF-1 (Zezulak and Green, 1986). The IGF-1 level is altered in AD patients’ brain, suggesting the effect of disrupted signaling of IGF-1 in AD. Besides, IGF-1 polymorphism rs972936 is associated with the high prevalence of AD (Vargas et al., 2011). Furthermore, IGF-1-mediated signals are implicated in tau phosphorylation, the cleavage of APP, transport of Aβ, memory formation, etc (Freude et al., 2009). IGF-1 prevents autophagy in human colorectal carcinoma cells (Wang and Gu, 2018), but the role of IGF-1 in the regulation of autophagy remains to be elucidated.

### Nerve Growth Factor (NGF)

Nerve growth factor is an emerging neurotrophin in adipose tissue biology. It is expressed in both WAT and BAT of rodents that were fed with a high-fat diet. Furthermore, both neurotrophins were found to have roles in the pathogenesis of other metabolic diseases, including cardiovascular diseases (Sornelli et al., 2009). In adipocytes, NGF is involved in energy homeostasis by regulating glucose and lipid metabolism (Chaldakov et al., 2003). Furthermore, NGF also plays a crucial role in neural disorders. In particular, the activity of NGF is elevated in the frontal and occipital cortex of AD patients. Measurement of the quantity of NGF is used as a diagnostic criterion for AD (Crutcher et al., 1993). Though NGF has been shown to activate autophagy in Schwann cells (Li et al., 2020), its role in regulating autophagy in brain cells need to be established.

### Tissue Factor (TF)

Tissue factor genes are expressed in the adipose tissue of obese rodents (Samad et al., 1998) and atherosclerotic plaque of humans (Pfeiffer and Schatz, 1995). TF is a cell-surface receptor that activates factor VII and initiates the coagulation cascade in the cardiovascular system (Edgington et al., 1991). TF is expressed in the brain parenchyma, neurons, astrocytes, and in the cortex. However, the immunoreactivity of TF was prominent in senile plaques (Faulk et al., 1990). TF also activates the coagulation factor in the brain by forming a complex with factor VII via the extrinsic pathway (Fleck et al., 1990; McComb et al., 1991). Further insight into TF would help to develop treatment/diagnostic strategies for AD. However, the role of TF in autophagy is not studied.

### Transforming Growth Factor Beta (TGF-β)

Transforming growth factor beta is a multi-functional growth factor and plays a crucial role in the determination and differentiation of mesenchymal progenitors in the adipogenic pathway (Fujimoto et al., 2003; Sato et al., 2009). Specifically, TGF-β inhibits adipogenesis through the smad3-dependent pathway (Choy and Derynck, 2003; Tsurutani et al., 2011). TGF-β1 is a candidate gene for AD, and its overexpression induces the deposition of the Aβ peptide (Mattson et al., 1997). Besides, TGF-β1 is involved in cellular responses following brain injury, and its level is found to be elevated in AD patients (Gómez-Pinilla and Cotman, 1993). Furthermore, overexpression of TGF-β1 triggers Aβ accumulation in senile plaques of AD patients (Luedecking et al., 2000). TGF-β is a potent inducer of autophagy in other cells, but its role in AD remains elusive. Altogether, this evidence emphasizes the importance of TGF-β and helps understanding the downstream signaling pathways of TGF-β in AD to strategize further therapeutic intervention.

### Vascular Endothelial Cell Growth Factor (VEGF)

Vascular endothelial cell growth factor helps in the expansion and lifelong growth of adipose tissues (Cao, 2007; Lijnen, 2007). Concomitant neovascularization parallel to the expansion of adipocytes is required for the efficient delivery of oxygen and nutrients. Adipose tissues release a variety of angiogenic growth factors, including VEGF, FGFs, etc., for neovascularization (Cao, 2007). Angiogenic suppression is one of the promising therapeutic approaches for the effective treatment of obesity and diabetes. Angiogenic inhibitors have been shown to possess promising efficacy in individuals with metabolic syndrome (Rupnick et al., 2002; Cao, 2010). It is well known that hypoxia leads to obesity due to inadequate angiogenesis, which is perhaps caused by the impaired VEGF signaling in the adipose tissue. However, the role of VEGF in obesity is unclear (Sung et al., 2013). Hypoxia also results in AD due to pathological alteration of the vasculature, which is one of the key features of AD, leading to the accumulation of atherosclerotic plaques and Aβ in arterial blood vessels of AD patients. However, the role of Aβ in vascularization is poorly understood. The levels of Aβs 40 and 42 in brain arterioles were found to be elevated in AD patients and transgenic AD mouse model. VEGF inhibits Aβ-induced endothelial apoptosis *in vitro*. In line with the above finding, neuron-specific expression of VEGF in transgenic mice restored memory in rodent model of AD (Religa et al., 2013). Evidence has suggested that VEGF expression is increased in response to autophagy induction (Yao et al., 2020). The exact relationship between VEGF and autophagy in AD remains to be elucidated. However, the above findings suggest that improvement of vascular functions would be a novel approach for the treatment of AD.

## Immunomodulatory Proteins

### Monocyte Chemotactic Protein-1 (MCP -1)

Monocyte chemotactic protein-1 belongs to the chemokine family. MCP-1 is involved in the remodeling and expansion of adipose tissue during the early stages of obesity (Low et al., 2001). Therefore, the disruption of MCP-1 expression is considered to be important in the alteration of the function and metabolism of adipose tissue, especially during the transition between lean and obese states (Sartipy and Loskutoff, 2003). Besides, MCP-1 recruits monocytes, thereby initiating and maintaining the inflammatory reactions in the adipose tissue (Sartipy and Loskutoff, 2003). MCP-1 also influences glucose and lipid metabolism (Loskutoff and Samad, 1998). In the CNS, MCP-1 is produced by astrocytes (Hurwitz et al., 1995) and microglia (Ishizuka et al., 1997). Sokolova et al. (2009) identified MCP-1 as the major predictor of AD, and they found consistent upregulation of MCP-1 in AD brain tissue. They further localized MCP-1 in neurons, astrocytes, and within plaques through immunohistochemistry (Sokolova et al., 2009). Yet, the involvement of MCP-1 in autophagy needs to be investigated further.

### C-Reactive Protein (CRP)

In adipose tissue, pro-inflammatory cytokines, such as IL-6 (Hotamisligil et al., 1995) and TNF alpha (Heinrich et al., 1990), stimulate the secretion of CRP (Warren et al., 1987). In particular, CRP levels are found to be elevated in visceral adipose tissue (Saijo et al., 2004). In diagnostics, CRP is used as a marker of inflammation (Stefanska et al., 2011). Inflammation, on the other hand, is crucial in cognitive decline, AD, and vascular dementia. Positively, a plethora of studies have linked AD with CRP levels. Furthermore, CRP was also found in both neurofibrillary tangles and senile plaques of AD patients (Engelhart et al., 2004). O’Bryant et al. (2013) found a decreased level of CRP among AD patients and suggested the rise in CRP is dependent on the progression of AD (O’Bryant et al., 2013). Another study, however, found no significant association between plasma CRP at the baseline and subsequent cognitive decline. Therefore, it was concluded that reduced levels of plasma CRP can be used as a biomarker to diagnose AD (Yarchoan et al., 2013). Though the data from the literature is contradicting, it needs to be further investigated. In the autophagic perspective, CRP is found to have a negative correlation with autophagy in kidney injury in mice (Bian et al., 2017). However, the role of CRP in regulating neuronal autophagy is unclear and needs to be explored.

### Serum Amyloid A (SAA) Proteins

Serum amyloid A proteins are mediators of metabolism and inflammation, and they are therefore correlated with the metabolic syndromes (Lin et al., 2001; Yang et al., 2006). Hitherto, four functional isoforms of SAA (SAA 1–4) have been identified in mice (Uhlar and Whitehead, 1999), among which SAA3 is predominantly expressed in the adipose tissue (Meek and Benditt, 1986; Reigstad et al., 2009). Scheja et al. (2008) found SAA3 upregulation in the adipose tissue of high-fat-fed mice and suggested SAA3 as a mediator of chronic inflammation and insulin resistance (Scheja et al., 2008). SAA3 is also found on the myelin sheath and underlying axonal membrane of cortical projection neurons, where large quantities of cholesterol are present. This leads to amyloidosis in the brain that eventually causes chronic inflammation and promotes AD (Chung et al., 2000). Furthermore, the systemic inflammation was suggested to increase the SAA proteins and found to enhance the amyloid deposition in the brain (Guo et al., 2002). SAA proteins, however, were never found to be associated with neuronal autophagy.

## Chemokines

### Chemerin

Chemerin is a chemoattractant protein and adipokine (Wittamer et al., 2003), secreted by the adipose tissue (Ernst and Sinal, 2010), which appears to promote adipocyte differentiation (Jialal et al., 2013). Several studies have shown the increased chemerin expression during adipogenesis in 3T3 L1 adipocytes (Bozaoglu et al., 2007; Goralski et al., 2007). In contrast, Sell et al. (2009) reported that chemerin mediates a negative crosstalk between adipose tissue and skeletal muscle, thereby contributing to the negative relationship between obesity and insulin sensitivity (Sell et al., 2009). Chemerin also serves as a ligand for CMKLR1 (chemokine-like receptor 1) (Kulig et al., 2011). Peng et al. (2015) reported a higher mRNA expression of CMKLR1 in the brain of AD patients and mice in response to systemic administration of LPS (Peng et al., 2015). In transgenic mice (AβPP/PS1), CMKLR1 and Aβ42 are co-localized in the hippocampus and cortex. There was a specific interaction between Aβ42 and CMKLR1 in rat basophilic leukemia (RBL) cells, which suggest CMKLR1 as a receptor for Aβ42. Furthermore, Aβ42 stimulates CMKLR1-RBL cells and primary glial cells to internalize the Aβ42-CMKLR1 complex, which helps in the clearance of Aβ42 (Kulig et al., 2011). Very recently, chemerin was reported to induce autophagy in bovine mammary epithelial cells (Hu et al., 2019). Therefore, it is reasonable to speculate that it might induce autophagy in microglial cells also, which needs to be explored.

### Regulated Upon Activation, Normally T Cell Expressed and Secreted (RANTES)

Regulated upon activation, normally T cell expressed and secreted, otherwise known as chemokine ligand 5 (CCL5), is a pro-inflammatory chemokine strongly involved in inflammation (Fischer et al., 2001). Wu et al. (2007) observed an increase in RANTES in WAT of obese mice and humans. In morbidly obese cases, RANTES expression was higher in visceral adipose tissue compared to subcutaneous adipose tissue (Wu et al., 2007). However, physical exercise was suggested to overcome the deleterious effects of RANTES-associated obesity (Baturcam et al., 2014). RANTES induces neuroinflammation and promotes the onset of neurodegenerative diseases, such as multiple sclerosis, AD, Parkinson’s disease (PD), and HIV-associated dementia (Galimberti et al., 2006). Kester et al. (2012) reported decreased expression of RANTES mRNA in the blood of AD patients (Kester et al., 2012). Tripathy et al. (2010) revealed RANTES upregulation in cerebral microcirculation of AD patients. They further confirmed that the treatment of neurons *in vitro* with RANTES offered a neuroprotective effect. Therefore, they suggested using RANTES to develop a novel therapeutic strategy for AD (Tripathy et al., 2010). Even though RANTES was proven to be involved in AD, its role in autophagy has not been established so far.

## Proteins Affecting the Fibrinolytic System

### Plasminogen Activator Inhibitor 1 (PAI-1)

Plasminogen activator inhibitor 1 is, in part, secreted by the adipose tissue (Alessi et al., 2007). PAI-1 is increased in obese humans and found to be decreased during weight loss. It is also associated with type-2 diabetes as a potential circulating marker. However, its role in obesity is not fully revealed (Alessi et al., 2007). In the AD mice model, the level of PAI-1 was increased (Kutz et al., 2012). Melchor et al. (2003) reported that the tissue plasminogen activator (tPA)-plasmin system plays a vital role in the degradation of Aβ *in vivo*. In AD patients, the chronic elevation of Aβ peptide in the brain correlates with the increased level of PAI-1 and inhibition of tPA–plasmin system. The tPA-plasminogen proteolytic cascade increases Aβ degradation and prevents Aβ-induced neurodegeneration (Melchor et al., 2003). This indicates that PAI-1 inhibits the plasmin activity and results in increased accumulation of Aβ. Positively, PAI-1 inhibitors were also suggested to use for therapeutic approaches against AD (Skrzypczak-Jankun and Jankun, 2010; Kutz et al., 2012). Conversely, the function of PAI-1 is not established in neuronal autophagy.

## Complement and Complement-Related Proteins

### Complement Factor B (CFB)

Complements are part of innate immunity and play crucial roles in assisting the antibodies and phagocytic cells to eliminate pathogens. It was observed that adipose tissue explants of obese patients were able to produce complement factors B, D, and C3 (Fain et al., 2004). CFB when overexpressed in the 3T3-L1 cell line, the genes associated with adipocytes differentiation/maturation were also enhanced. This proves the efficacy of CFB in adipocyte differentiation (Matsunaga et al., 2018). Interestingly, a study by Manral et al. (2012) compared the CSF samples of AD patients and control and revealed the differential expression of CFB. This advocates CFB as a marker for AD (Manral et al., 2012). A very recent meta-analysis study showed that the concentration of CFB was lowered in AD (Krance et al., 2019). However, the evidence is lacking about CFB in inducing autophagy.

### Complement Factor H (CFH)

The expression of CFH was significantly increased in the subcutaneous fat of humans. Furthermore, the expression was also highly correlated with insulin resistance (Moreno-Navarrete et al., 2010). In a study by Thambisetty et al. (2008), the plasma CFH level was increased up to 13-fold in AD patients compared to control patients (Thambisetty et al., 2008). The CFH was also found to be accumulated in the amyloid plaque. Toledo et al. (2014), however, indicated that CFH is not a suitable biomarker for AD (Toledo et al., 2014). The current understanding of the role of CFH in autophagy is limited and needs to be established.

## Proteins in Metabolic Process

### Adiponectin

Adiponectin is one of the cytokines produced by adipose tissue. It is involved in many physiological processes, such as insulin sensitivity, glucose homeostasis, and fatty acid catabolism (Dzielińska et al., 2003; Ouchi et al., 2003). Adiponectin levels are inversely proportional to the adipose tissue mass, and they are therefore found to be at low concentrations during obesity. The role of adiponectin in cognition and brain activity remains controversial. For instance, Une et al. (2011) showed that elevated adiponectin levels were associated with AD (Une et al., 2011). In contrast, van Himbergen et al. (2012), in a population-based study, showed an elevated level of adiponectin in dementia-free individuals (van Himbergen et al., 2012). On the other hand, Gu et al. (2010) found no significant association between adiponectin levels and the risk of cognitive decline and dementia (Gu et al., 2010). A similar observation was made by Bigalke et al. (2011). This view was further supported by other researchers as well. Teixeira et al. (2013) inferred that there is no correlation between adiponectin levels and mild cognitive impairment, and AD (Teixeira et al., 2013). Adiponectin has been proven to induce autophagy in skeletal muscle and reduce insulin sensitivity (Liu et al., 2015). However, the role of adiponectin in the modulation of neuronal autophagy is still unclear and needs to be explored.

### Cholesteryl Ester Transfer Protein (CETP)

Adipose tissue majorly contributes to the secretion of CETP, which, in turn, helps adipocytes in the accumulation of cholesteryl ester from the HDL of a dietary source (Radeau et al., 1998). CETP concentration is found to be elevated during hyperlipoproteinemic states. This implies the enhanced delivery of lipoproteins to adipose tissue and increased CETP gene expression in adipocytes (Radeau et al., 1995). The brain, in part, also synthesizes CETP, which might play an important role in the transport and redistribution of cholesterol within the brain, possibly by enhancing the neuronal uptake of HDL particles. Furthermore, it has also been proven that cholesterol positively regulates the production of Aβ (Rodríguez et al., 2006). Cholesterol, on the other hand, was also reported to impair Aβ degradation, eventually culminating its role in autophagy (Barbero-Camps et al., 2018). Sanders et al. (2010) found that single nucleotide polymorphism (SNP) at CETP codon 405 homozygosity is associated with slower memory decline and a lower incidence of dementia and AD risk (Sanders et al., 2010). Therefore, it is reasonable to speculate CETP as a causative agent of AD. However, the role of CETP in autophagy is not established.

### Lipoprotein Lipase (LPL)

Lipoprotein lipase plays a crucial role in the physiology of adipocytes because it majorly contributes to the production of fatty acids. In general, lipoprotein particles transported out of the vasculature system contribute to a significant source of fatty acids for adipocyte storage (Mead et al., 2002). Apart from adipose and muscle, LPL is also secreted by the brain. In mouse primary astrocytes, LPL binds to Aβ and promotes cell-surface association and uptake of Aβ (Nishitsuji et al., 2011). LPL is thus a novel Aβ-binding protein that promotes cellular uptake and degradation of Aβ. LPL is also co-localized with senile plaques in the brains of AD patients, and if there are any mutations they are associated with the severity of pathophysiological features of AD (Gonzales and Orlando, 2007). The role of LPL in autophagy has not been reported.

### Retinol Binding Protein 4 (RBP4)

In adipocytes, systemic glucose metabolism is regulated by the release of serum RBP4 (Yang et al., 2005). Therefore, RBP4 overexpression causes insulin resistance, while the reduced expression ameliorates insulin resistance. Furthermore, elevated levels of RBP4 were associated with type 2 diabetes. Altogether, adipocytes utilize RBP4 to regulate whole-body insulin sensitivity. The presence of cellular retinol-binding protein 1 (CRBP1) and cellular retinoic acid-binding protein 1 (CRABP1) was confirmed in the dendritic layers and dentate gyrus (Shudo et al., 2009). Retinoid signaling is pivotal in the brain and is suggested to have a physiological role in hypothalamus, amygdala, hippocampus, etc. This underscores the importance of retinoid signaling and RBP4 in AD (Lane and Bailey, 2005). To date, however, there was no evidence available on RBP4 in the regulation of autophagy.

### Resistin

Resistin is an adipokine, which hostile insulin action. Resistin is upregulated with genetic forms of obesity and downregulated with anti-diabetic drugs (Letra et al., 2014). In postmenopausal women, increased resistin mRNA expression causes insulin resistance and culminates in obesity-related disorders (Sadashiv et al., 2012). Lu et al. (2013) attributed neuroprotective effects to resistin due to its efficacy in acting against Aβ deposition. Resistin inhibits AD, perhaps by decreasing the level of reactive oxygen species, improving the mitochondrial function, and preventing apoptosis (Lu et al., 2013). Resistin was recently suggested as a potential biomarker to diagnose AD (Yu et al., 2018). In the SH-SY5Y human neuroblastoma cell line, resistin inhibits autophagy through the repression of autophagic markers. In wild-type mice, resistin treatment was found to inhibit the mRNA expression of autophagy markers. This suggests resistin as a potential inhibitor of autophagy (Miao et al., 2018).

## Enzymes for Steroid Metabolism

### 11β-Hydroxysteroid Dehydrogenase Type 1 (11β-HSD1)

11β-HSD1 generates active glucocorticoids (cortisol and corticosterone) that are strong inhibitors of angiogenesis. 11β-HSD1 supports the increased expression of angiogenic factors such as VEGF, TGF-β, and HGF, which are directly involved in the augmentation of adipose tissue. Thus, it is prudent to consider the role of 11β-HSD1 in adipose tissue dysfunction during obesity and angiogenesis (Lee et al., 2013). Age-related cognition was found to be declined upon chronic exposure to glucocorticoids, and this is inhibited by the 11β-HSD1 inhibitor (UE2316). Treatment with UE2316 consequently decreased the number of β-amyloid plaques and improved the memory in the AD mice model (Sooy et al., 2015). Furthermore, selective inhibitors of 11β-HSD1 also improved the memory and ameliorated the condition of AD in aged rodents (Mohler et al., 2011). Therefore, inhibition of 11β-HSD1 would be a novel strategy for the treatment of age-related cognitive disorders. Interestingly, another potent inhibitor of 11β-HSD1, RL-118, improved autophagy markers such as Beclin1, light chain 3B (LC3B), AMP-activated protein kinase alpha (AMPK α), and mammalian target of rapamycin (mTOR), which signified the role of 11β-HSD1 in autophagy. Additionally, RL-118 was proven to prevent neuroinflammation and cognitive decline associated with AD (Puigoriol-Illamola et al., 2018). 11β-HSD1 is thus a key player associated with adipose tissue, AD, and autophagy. Therefore, we suggest it as a potential target to manage the complications.

### 17β-Hydroxysteroid Dehydrogenase (17β-HSD)

Adipose tissues from rats and humans express 17β-HSD, which is involved in the peripheral synthesis of androgens and estrogens (Martel et al., 1992). It was also reported that 17β-HSD binds with the β-amyloid peptide (He et al., 1999). The high levels of 17β-HSD abolished the steroid hormone homeostasis in synapses and eventually lead to the loss of synapses in the hippocampus of the AD mouse model (He et al., 1999). The role of 17β-HSD has never been explored in autophagy.

### Cytochrome P450 Enzymes

Cytochrome P450 is induced in the WAT of rats (Ellero et al., 2010). The brain also secretes low levels of cytochrome P450 in various isoforms. These enzymes are likely to be involved in neurosteroid synthesis, the metabolism of drugs, and the protection of the brain from toxins (Nicholson and Renton, 1999). Cytochrome P450 46A1 (CYP46A1) helps in the clearance of cholesterol from the brain. Increased CYP46A1 expression in mice improves cognition and decreases the manifestations of AD (Mast et al., 2014). CYP46A1 could, therefore, be exploited for its beneficial role in AD. However, the role of cytochrome p450 has not been studied in autophagy.

## Conclusion and Future Direction

Recent evidences have pointed out that obesity is a predisposing factor for the development of AD and there is a strong association between dementia and adiposity. The current review advocates the possible correlation between the expressions of biomolecules secreted by adipose tissue with dysregulation in neurons thereby leading to progression toward AD. Obesity-mediated signaling may evoke AD by secreting a variety of signaling molecules, which includes cytokines, growth factors, immunomodulatory protein, complement and complement- related proteins, steroidogenic enzymes, etc. The precise mechanism that promotes AD through factors secreted by adipose tissue, however, remains elusive. It is well established that autophagy prevents the development of AD. Surprisingly, the roles of a number of adipose-derived molecules in autophagy, especially in microglial cells, have not been studied in detail, and study into this area might thus provide vital clues.

## Author Contributions

PP, SAr, DS, and SAc conceived the idea and wrote the manuscript. SAc edited the manuscript. PP, DS, and SAr developed the idea and edited the manuscript to the final form.

## Conflict of Interest

The authors declare that the research was conducted in the absence of any commercial or financial relationships that could be construed as a potential conflict of interest.

## References

[B1] Alessi M.-C. Poggi M. Juhan-Vague I. (2007). Plasminogen activator inhibitor-1, adipose tissue and insulin resistance. *Curr. Opin. Lipidol.* 18 240–245. 10.1097/mol.0b013e32814e6d29 17495595

[B2] Alzheimer’s Association (2006). Early-onset dementia: a national challenge, a future crisis. *Alzheimer’s Assoc*. 35 263–281.

[B3] An Y. A. Sun K. Joffin N. Zhang F. Deng Y. Donze O. (2017). Angiopoietin-2 in white adipose tissue improves metabolic homeostasis through enhanced angiogenesis. *eLife* 6:e24071. 10.7554/eLife.24071 28355132 PMC5391203

[B4] Azuma K. Katsukawa F. Oguchi S. Murata M. Yamazaki H. Shimada A. (2003). Correlation between serum resistin level and adiposity in obese individuals. *Obes. Res.* 11 997–1001. 10.1038/oby.2003.137 12917505

[B5] Bacher M. Deuster O. Aljabari B. Egensperger R. Neff F. Jessen F. (2010). The role of macrophage migration inhibitory factor in Alzheimer’s disease. *Mol. Med.* 16 116–121. 20200619 10.2119/molmed.2009.00123 PMC2829616

[B6] Barbero-Camps E. Roca-Agujetas V. Bartolessis I. de Dios C. Fernández-Checa J. C. Marí M. (2018). Cholesterol impairs autophagy-mediated clearance of amyloid beta while promoting its secretion. *Autophagy* 14 1129–1154. 10.1080/15548627.2018.1438807 29862881 PMC6103708

[B7] Baturcam E. Abubaker J. Tiss A. Abu-Farha M. Khadir A. Al-Ghimlas F. (2014). Physical exercise reduces the expression of RANTES and its CCR5 receptor in the adipose tissue of obese humans. *Media. Inflam.* 2014 627150. 10.1155/2014/627150 24895488 PMC4016945

[B8] Benoit S. C. Clegg D. J. Seeley R. J. Woods S. C. (2004). Insulin and leptin as adiposity signals. *Recent Prog. Hormone Res.* 59 267–286. 14749506 10.1210/rp.59.1.267

[B9] Bian A. Shi M. Flores B. Gillings N. Li P. Yan S. X. (2017). Downregulation of autophagy is associated with severe ischemia-reperfusion-induced acute kidney injury in overexpressing C-reactive protein mice. *PLoS One* 12:e0181848. 10.1371/journal.pone.0181848 28886014 PMC5590740

[B10] Bigalke B. Schreitmüller B. Sopova K. Paul A. Stransky E. Gawaz M. (2011). Adipocytokines and CD34+ progenitor cells in Alzheimer’s disease. *PLoS One* 6:e20286. 10.1371/journal.pone.0020286 21633502 PMC3102092

[B11] Bossù P. Ciaramella A. Salani F. Vanni D. Palladino I. Caltagirone C. (2010). Interleukin-18, from neuroinflammation to Alzheimer’s disease. *Curr. Pharm. Design* 16 4213–4224. 10.1111/bpa.12478 21184660

[B12] Bozaoglu K. Bolton K. McMillan J. Zimmet P. Jowett J. Collier G. (2007). Chemerin is a novel adipokine associated with obesity and metabolic syndrome. *Endocrinology* 148 4687–4694. 10.1210/en.2007-0175 17640997

[B13] Cao Y. (2007). Angiogenesis modulates adipogenesis and obesity. *J. Clin. Invest.* 117 2362–2368. 10.1172/jci32239 17786229 PMC1963348

[B14] Cao Y. (2010). Adipose tissue angiogenesis as a therapeutic target for obesity and metabolic diseases. *Nat. Rev. Drug Discov.* 9 107–115. 10.1038/nrd3055 20118961

[B15] Chakrabarty P. Tianbai L. Herring A. Ceballos-Diaz C. Das P. Golde T. E. (2012). Hippocampal expression of murine IL-4 results in exacerbation of amyloid deposition. *Mol. Neurodegen.* 7:36. 10.1186/1750-1326-7-36 22838967 PMC3441281

[B16] Chaldakov G. N. Stankulov I. S. Hristova M. Ghenev P. I. (2003). Adipobiology of disease: adipokines and adipokine-targeted pharmacology. *Curr. Pharm. Design* 9 1023–1031. 10.2174/1381612033455152 12678860

[B17] Choy L. Derynck R. (2003). Transforming growth factor-β inhibits adipocyte differentiation by Smad3 interacting with CCAAT/enhancer-binding protein (C/EBP) and repressing C/EBP transactivation function. *J. Biol. Chem.* 278 9609–9619. 10.1074/jbc.m212259200 12524424

[B18] Chu S.-H. Feng D.-F. Ma Y.-B. Zhu Z.-A. Zhang H. Qiu J.-H. (2009). Stabilization of hepatocyte growth factor mRNA by hypoxia-inducible factor 1. *Mol. Biol. Rep.* 36 1967–1975. 10.1007/s11033-008-9406-1 18979225

[B19] Chung T.-F. Liang J.-S. Schreiber B. M. Sipe J. D. McKee A. Fine R. E. (2000). Serum amyloid A in Alzheimer’s disease brain is predominantly localized to myelin sheaths and axonal membrane. *Amyloid* 7 105–110. 10.3109/13506120009146246 10842712

[B20] Cohen B. Barkan D. Levy Y. Goldberg I. Fridman E. Kopolovic J. (2001). Leptin induces angiopoietin-2 expression in adipose tissues. *J. Biol. Chem.* 276 7697–7700. 10.1074/jbc.c000634200 11152449

[B21] Coppack S. W. (2001). Pro-inflammatory cytokines and adipose tissue. *Proc. Nutr. Soc.* 60 349–356. 10.1079/pns2001110 11681809

[B22] Crutcher K. A. Scott S. A. Liang S. Everson W. V. Weingartner J. (1993). Detection of NGF-like activity in human brain tissue: increased levels in Alzheimer’s disease. *J. Neurosci.* 13 2540–2550. 10.1523/jneurosci.13-06-02540.1993 8501520 PMC6576515

[B23] Dallabrida S. M. Zurakowski D. Shih S.-C. Smith L. E. Folkman J. Moulton K. S. (2003). Adipose tissue growth and regression are regulated by angiopoietin-1. *Biochem. Biophys. Res. Commun.* 311 563–571. 10.1016/j.bbrc.2003.10.007 14623307

[B24] de Courten B. V. Degawa-Yamauchi M. Considine R. V. Tataranni P. A. (2004). High serum resistin is associated with an increase in adiposity but not a worsening of insulin resistance in Pima Indians. *Diabetes* 53 1279–1284. 10.2337/diabetes.53.5.1279 15111497

[B25] Dickson D. W. (1997). The pathogenesis of senile plaques. *J. Neuropathol. Exp. Neurol.* 56 321–339. 10.1097/00005072-199704000-00001 9100663

[B26] Dzielińska Z. Januszewicz A. Więcek A. Demkow M. Makowiecka-Cieśla M. Prejbisz A. (2003). Decreased plasma concentration of a novel anti-inflammatory protein—adiponectin—in hypertensive men with coronary artery disease. *Thromb. Res.* 110 365–369. 10.1016/j.thromres.2003.08.004 14592564

[B27] Edgington T. S. Mackman N. Brand K. Ruf W. (1991). The structural biology of expression and function of tissue factor. *Thromb. Haemost.* 65 67–79. 1833852

[B28] Elias M. F. Elias P. K. Sullivan L. M. Wolf P. A. D’agostino R. B. (2003). Lower cognitive function in the presence of obesity and hypertension: the Framingham heart study. *Int. J. Obes.* 27 260–268. 10.1038/sj.ijo.802225 12587008

[B29] Ellero S. Chakhtoura G. Barreau C. Langouet S. Benelli C. Penicaud L. (2010). Xenobiotic-metabolizing cytochromes p450 in human white adipose tissue: expression and induction. *Drug Metab. Dispos.* 38 679–686. 10.1124/dmd.109.029249 20035023

[B30] Engelhart M. J. Geerlings M. I. Meijer J. Kiliaan A. Ruitenberg A. van Swieten J. C. (2004). Inflammatory proteins in plasma and the risk of dementia: the rotterdam study. *Arch. Neurol.* 61 668–672. 15148142 10.1001/archneur.61.5.668

[B31] Ernst M. C. Sinal C. J. (2010). Chemerin: at the crossroads of inflammation and obesity. *Trends Endocrinol. Metab.* 21 660–667. 10.1016/j.tem.2010.08.001 20817486

[B32] Fain J. N. (2006). Release of interleukins and other inflammatory cytokines by human adipose tissue is enhanced in obesity and primarily due to the nonfat cells. *Vitam. Hormon.* 74 443–477. 10.1016/s0083-6729(06)74018-3 17027526

[B33] Fain J. N. Madan A. K. Hiler M. L. Cheema P. Bahouth S. W. (2004). Comparison of the release of adipokines by adipose tissue, adipose tissue matrix, and adipocytes from visceral and subcutaneous abdominal adipose tissues of obese humans. *Endocrinology* 145 2273–2282. 10.1210/en.2003-1336 14726444

[B34] Faulk W. P. Labarrere C. A. Carson S. D. (1990). Tissue factor: identification and characterization of cell types in human placentae. *Blood* 76 86–96. 10.1182/blood.v76.1.86.bloodjournal76186 2364176

[B35] Feldmann M. Maini R. N. (2003). TNF defined as a therapeutic target for rheumatoid arthritis and other autoimmune diseases. *Nat. Med.* 9 1245–1250. 10.1038/nm939 14520364

[B36] Fischer F. R. Luo Y. Luo M. Santambrogio L. Dorf M. E. (2001). RANTES-induced chemokine cascade in dendritic cells. *J. Immunol.* 167 1637–1643. 10.4049/jimmunol.167.3.1637 11466387

[B37] Fleck R. A. Rao L. V. M. Rapaport S. I. Varki N. (1990). Localization of human tissue factor antigen by immunostaining with monospecific, polyclonal anti-human tissue factor antibody. *Thromb. Res.* 57 765–781. 10.1016/0049-3848(90)90034-a 2237820

[B38] François A. Terro F. Janet T. Bilan A. R. Paccalin M. Page G. (2013). Involvement of interleukin-1β in the autophagic process of microglia: relevance to Alzheimer’s disease. *J. Neuroinflam.* 10 915. 10.1186/1742-2094-10-151 PMC3878742 24330807

[B39] Freude S. Schilbach K. Schubert M. (2009). The role of IGF-1 receptor and insulin receptor signaling for the pathogenesis of Alzheimer’s disease: from model organisms to human disease. *Curr. Alzheimer Res.* 6 213–223. 10.2174/156720509788486527 19519303

[B40] Fujimoto M. Maezawa Y. Yokote K. Joh K. Kobayashi K. Kawamura H. (2003). Mice lacking Smad3 are protected against streptozotocin-induced diabetic glomerulopathy. *Biochem. Biophys. Res. Commun.* 305 1002–1007. 10.1016/s0006-291x(03)00885-4 12767930

[B41] Galimberti D. Schoonenboom N. Scheltens P. Fenoglio C. Bouwman F. Venturelli E. (2006). Intrathecal chemokine synthesis in mild cognitive impairment and Alzheimer disease. *Arch. Neurol.* 63 538–543. 16606766 10.1001/archneur.63.4.538

[B42] Gómez-Pinilla F. Cotman C. W. (1993). Transforming growth factor-beta 1 is in plaques in Alzheimer and Down pathologies. *Neuroreport* 4 69–72. 10.1097/00001756-199301000-00018 8453039

[B43] Gonzales A. M. Orlando R. A. (2007). Role of adipocyte-derived lipoprotein lipase in adipocyte hypertrophy. *Nutr. Metab.* 4 22. 10.1186/1743-7075-4-22 17971230 PMC2174487

[B44] Goralski K. B. McCarthy T. C. Hanniman E. A. Zabel B. A. Butcher E. C. Parlee S. D. (2007). Chemerin, a novel adipokine that regulates adipogenesis and adipocyte metabolism. *J. Biol. Chem.* 282 28175–28188. 10.1074/jbc.m700793200 17635925

[B45] Gu Y. Luchsinger J. A. Stern Y. Scarmeas N. (2010). Mediterranean diet, inflammatory and metabolic biomarkers, and risk of Alzheimer’s disease. *J. Alzheimer’s Dis.* 22 483–492. 10.3233/JAD-2010-100897 20847399 PMC3022949

[B46] Guo J. Yu J. Grass D. de Beer F. C. Kindy M. S. (2002). Inflammation-dependent cerebral deposition of serum amyloid a protein in a mouse model of amyloidosis. *J. Neurosci.* 22 5900–5909. 10.1523/jneurosci.22-14-05900.2002 12122052 PMC6757908

[B47] Gustafson D. Rothenberg E. Blennow K. Steen B. Skoog I. (2003). An 18-year follow-up of overweight and risk of Alzheimer disease. *Arch. Inter. Med.* 163 1524–1528. 12860573 10.1001/archinte.163.13.1524

[B48] He X.-Y. Merz G. Mehta P. Schulz H. Yang S.-Y. (1999). Human Brain Short Chain l-3-Hydroxyacyl Coenzyme A Dehydrogenase Is a Single-domain Multifunctional Enzyme CHARACTERIZATION OF A NOVEL 17β-HYDROXYSTEROID DEHYDROGENASE. *J. Biol. Chem.* 274 15014–15019. 10.1074/jbc.274.21.15014 10329704

[B49] Hebert L. E. Weuve J. Scherr P. A. Evans D. A. (2013). Alzheimer disease in the United States (2010–2050) estimated using the 2010 census. *Neurology* 80 1778–1783. 10.1212/wnl.0b013e31828726f5 23390181 PMC3719424

[B50] Heinrich P. C. Castell J. V. Andus T. (1990). Interleukin-6 and the acute phase response. *Biochem. J.* 265 621–636. 10.1042/bj2650621 1689567 PMC1133681

[B51] Hoareau L. Bencharif K. Rondeau P. Murumalla R. Ravanan P. Tallet F. (2010). Signaling pathways involved in LPS induced TNFalpha production in human adipocytes. *J. Inflam.* 7:1. 10.1186/1476-9255-7-1 20148136 PMC2819999

[B52] Hotamisligil G. S. Arner P. Caro J. F. Atkinson R. L. Spiegelman B. M. (1995). Increased adipose tissue expression of tumor necrosis factor-alpha in human obesity and insulin resistance. *J. Clin. Invest.* 95 2409–2415. 10.1172/jci117936 7738205 PMC295872

[B53] Hotamisligil G. S. Shargill N. S. Spiegelman B. M. (1993). Adipose expression of tumor necrosis factor-alpha: direct role in obesity-linked insulin resistance. *Science* 259 87–91. 10.1126/science.7678183 7678183

[B54] Hu B. Song W. Tang Y. Shi M. Li H. Yu D. (2019). Induction of chemerin on autophagy and apoptosis in dairy cow mammary epithelial cells. *Animals* 9:E848. 10.3390/ani9100848 31640289 PMC6826480

[B55] Huang X. (2018). The potential role of HGF-MET signaling and autophagy in the war of Alectinib versus Crizotinib against ALK-positive NSCLC. *J. Exp. Clin. Cancer Res.* 37:33. 10.1186/s13046-018-0707-5 29463284 PMC5819222

[B56] Hurwitz A. A. Lyman W. D. Herman J. W. (1995). Tumor necrosis factor α and transforming growth factor β upregulate astrocyte expression of monocyte chemoattractant protein-1. *J. Neuroimmunol.* 57 193–198. 10.1016/0165-5728(95)00011-p 7706436

[B57] Ishizuka K. Kimura T. Igata-Yi R. Katsuragi S. Takamatsu J. Miyakawa T. (1997). Identification of monocyte chemoattractant protein-1 in senile plaques and reactive microglia of Alzheimer’s disease. *Psychiatry Clin. Neurosci.* 51 135–138. 10.1111/j.1440-1819.1997.tb02375.x 9225377

[B58] Itoh N. Ohta H. (2014). Roles of FGFs as adipokines in adipose tissue development, remodeling, and metabolism. *Front. Endocrinol.* 5:18. 10.3389/fendo.2014.00018 24605108 PMC3932445

[B59] Jequier E. (2002). Leptin signaling, adiposity, and energy balance. *Ann. N. Y. Acad. Sci.* 967 379–388. 10.1111/j.1749-6632.2002.tb04293.x 12079865

[B60] Jialal I. Devaraj S. Kaur H. Adams-Huet B. Bremer A. A. (2013). Increased chemerin and decreased omentin-1 in both adipose tissue and plasma in nascent metabolic syndrome. *J. Clin. Endocrinol. Metab.* 98 E514–E517. 23303213 10.1210/jc.2012-3673

[B61] Jin M. Wang F. Liu W. Qi D. Gu C. Mao C. J. (2018). A critical role of autophagy in regulating microglia polarization in neurodegeneration. *Front. Aging Neurosci.* 10:378. 10.3389/fnagi.2018.00378 30515090 PMC6256089

[B62] Juge-Aubry C. E. Somm E. Pernin A. Alizadeh N. Giusti V. Dayer J.-M. (2005). Adipose tissue is a regulated source of interleukin-10. *Cytokine* 29 270–274. 15749027 10.1016/j.cyto.2004.10.017

[B63] Katsouri L. Ashraf A. Birch A. M. Lee K. K. L. Mirzaei N. Sastre M. (2015). Systemic administration of fibroblast growth factor-2 (FGF2) reduces BACE1 expression and amyloid pathology in APP23 mice. *Neurobiol. Aging* 36 821–831. 10.1016/j.neurobiolaging.2014.10.004 25457554

[B64] Kern P. A. Ranganathan S. Li C. Wood L. Ranganathan G. (2001). Adipose tissue tumor necrosis factor and interleukin-6 expression in human obesity and insulin resistance. *Am. J. Physiol. Endocrinol. Metab.* 280 E745–E751. 11287357 10.1152/ajpendo.2001.280.5.E745

[B65] Kershaw E. E. Flier J. S. (2004). Adipose tissue as an endocrine organ. *J. Clin. Endocrinol. Metab.* 89 2548–2556. 15181022 10.1210/jc.2004-0395

[B66] Kester M. I. van der Flier W. M. Visser A. Blankenstein M. A. Scheltens P. Oudejans C. B. (2012). Decreased mRNA expression of CCL5 [RANTES] in Alzheimer’s disease blood samples. *Clin. Chem. Lab. Med.* 50 61–65. 10.1515/CCLM.2011.731 21942811

[B67] King A. Brain A. Hanson K. Dittmann J. Vickers J. Fernandez-Martos C. (2018). Disruption of leptin signalling in a mouse model of Alzheimer’s disease. *Metab. Brain Dis.* 33 1097–1110. 10.1007/s11011-018-0203-9 29546689

[B68] Kivipelto M. Ngandu T. Fratiglioni L. Viitanen M. Kåreholt I. Winblad B. (2005). Obesity and vascular risk factors at midlife and the risk of dementia and Alzheimer disease. *Arch. Neurol.* 62 1556–1560. 16216938 10.1001/archneur.62.10.1556

[B69] Koenen T. B. Stienstra R. Van Tits L. J. De Graaf J. Stalenhoef A. F. H. Joosten L. A. B. (2011). Hyperglycemia activates caspase-1 and TXNIP-mediated IL-1β transcription in human adipose tissue. *Diabetes* 60 517–524. 10.2337/db10-0266 21270263 PMC3028351

[B70] Krance S. H. Wu C.-Y. Zou Y. Mao H. Toufighi S. He X. (2019). The complement cascade in Alzheimer’s disease: a systematic review and meta-analysis. *Mol. Psychiatry* [Epub ahead of print]. 10.1038/s41380-019-0536-8 31628417

[B71] Kulig P. Kantyka T. Zabel B. A. Banaś M. Chyra A. Stefańska A. (2011). Regulation of chemerin chemoattractant and antibacterial activity by human cysteine cathepsins. *J. Immunol.* 187 1403–1410. 10.4049/jimmunol.1002352 21715684 PMC3140563

[B72] Kutz S. M. Higgins C. E. Higgins P. J. (2012). Novel combinatorial therapeutic targeting of PAI-1 (SERPINE1) gene expression in Alzheimer’s disease. *Mol. Med. Ther.* 1:106. 10.4172/2324-8769.1000106 PMC3703665 23847772

[B73] Lane M. A. Bailey S. J. (2005). Role of retinoid signalling in the adult brain. *Prog. Neurobiol.* 75 275–293. 10.1016/j.pneurobio.2005.03.002 15882777

[B74] Lashuel H. A. Aljabari B. Sigurdsson E. M. Metz C. N. Leng L. Callaway D. J. E. (2005). Amyloid fibril formation by macrophage migration inhibitory factor. *Biochem. Biophys. Res. Commun.* 338 973–980. 10.1016/j.bbrc.2005.10.040 16286092

[B75] Laterra J. Nam M. Rosen E. Rao J. S. Lamszus K. Goldberg I. D. (1997). Scatter factor/hepatocyte growth factor gene transfer enhances glioma growth and angiogenesis in vivo. *Lab. Invest.* 76 565–577. 9111517

[B76] Lee J. H. Gao Z. Ye J. (2013). Regulation of 11β-HSD1 expression during adipose tissue expansion by hypoxia through different activities of NF-κB and HIF-1α. *Am. J. Physiol. Endocrinol. Metab.* 304 E1035–E1041. 23512810 10.1152/ajpendo.00029.2013 PMC3651619

[B77] Letra L. Santana I. Seiça R. (2014). Obesity as a risk factor for Alzheimer’s disease: the role of adipocytokines. *Metab. Brain Dis.* 29 563–568. 10.1007/s11011-014-9501-z 24553879

[B78] Li L. Li Y. Zhao D. Jin M. Ni H. (2018). Leptin-regulated autophagy plays a role in long-term neurobehavioral injury after neonatal seizures and the regulation of zinc/cPLA2 and CaMK II signaling in cerebral cortex. *Epilepsy Res.* 146 103–111. 10.1016/j.eplepsyres.2018.07.023 30092488

[B79] Li R. Li D. Wu C. Ye L. Wu Y. Yuan Y. (2020). Nerve growth factor activates autophagy in Schwann cells to enhance myelin debris clearance and to expedite nerve regeneration. *Theranostics* 10 1649–1677. 10.7150/thno.40919 32042328 PMC6993217

[B80] Lieb W. Beiser A. S. Vasan R. S. Tan Z. S. Au R. Harris T. B. (2009). Association of plasma leptin levels with incident Alzheimer disease and MRI measures of brain aging. *JAMA* 302 2565–2572. 10.1001/jama.2009.1836 20009056 PMC2838501

[B81] Lijnen H. R. (2007). Angiogenesis and obesity. *Cardiovasc. Res.* 78 286–293. 10.1093/cvr/cvm007 18006485

[B82] Lin Y. Rajala M. W. Berger J. P. Moller D. E. Barzilai N. Scherer P. E. (2001). Hyperglycemia-induced production of acute phase reactants in adipose tissue. *J. Biol. Chem.* 276 42077–42083. 10.1074/jbc.m107101200 11546817

[B83] Liu Y. Palanivel R. Rai E. Park M. Gabor T. V. Scheid M. P. (2015). Adiponectin stimulates autophagy and reduces oxidative stress to enhance insulin sensitivity during high-fat diet feeding in mice. *Diabetes* 64 36–48. 10.2337/db14-0267 25071026

[B84] Loskutoff D. J. Samad F. (1998). The adipocyte and hemostatic balance in obesity: studies of PAI-1. *Arteriosc. Thromb. Vasc. Biol.* 18 1–6. 10.1161/01.atv.18.1.1 9445248

[B85] Low Q. E. H. Drugea I. A. Duffner L. A. Quinn D. G. Cook D. N. Rollins B. J. (2001). Wound healing in MIP-1α−/− and MCP−1−/− mice. *Am. J. Pathol.* 159 457–463. 10.1016/s0002-9440(10)61717-8 11485904 PMC1850564

[B86] Lu D. Chen J. Tan T. Huang C. Yeh W. Hsu H. (2013). Resistin protects against 6-hydroxydopamine-induced cell death in dopaminergic-like MES23. 5 cells. *J. Cell. Physiol.* 228 563–571. 10.1002/jcp.24163 22806254

[B87] Luchsinger J. A. Gustafson D. R. (2009). Adiposity and Alzheimer’s disease. *Curr. Opin. Clin. Nutr. Metab. Care* 12 15–21. 19057182 10.1097/MCO.0b013e32831c8c71 PMC2771208

[B88] Luedecking E. K. DeKosky S. T. Mehdi H. Ganguli M. Kamboh M. I. (2000). Analysis of genetic polymorphisms in the transforming growth factor-β1 gene and the risk of Alzheimer’s disease. *Hum. Genet.* 106 565–569. 10.1007/s004390050026 10914688

[B89] Manral P. Sharma P. Hariprasad G. Tripathi M. Srinivasan A. (2012). Can apolipoproteins and complement factors be biomarkers of Alzheimer’s disease? *Curr. Alzheimer Res.* 9 935–943. 10.2174/156720512803251110 22631439

[B90] Martel C. Rheaume E. Takahashi M. Trudel C. Couet J. Luu-The V. (1992). Distribution of 17β-hydroxysteroid dehydrogenase gene expression and activity in rat and human tissues. *J. Steroid Biochem. Mol. Biol.* 41 597–603. 10.1016/0960-0760(92)90390-5 1314080

[B91] Mast N. Li Y. Linger M. Clark M. Wiseman J. Pikuleva I. A. (2014). Pharmacologic stimulation of cytochrome P450 46A1 and cerebral cholesterol turnover in mice. *J. Biol. Chem.* 289 3529–3538. 10.1074/jbc.m113.532846 24352658 PMC3916554

[B92] Matsuda S. Saito H. Nishiyama N. (1990). Effect of basic fibroblast growth factor on neurons cultured from various regions of postnatal rat brain. *Brain Res.* 520 310–316. 10.1016/0006-8993(90)91720-2 2207638

[B93] Matsunaga H. Iwashita M. Shinjo T. Yamashita A. Tsuruta M. Nagasaka S. (2018). Adipose tissue complement factor B promotes adipocyte maturation. *Biochem. Biophys. Res. Commun.* 495 740–748. 10.1016/j.bbrc.2017.11.069 29137982

[B94] Mattson M. P. Barger S. W. Furukawa K. Bruce A. J. Wyss-Coray T. Mark R. J. (1997). Cellular signaling roles of TGFβ, TNFα and βAPP in brain injury responses and Alzheimer’s disease. *Brain Res. Rev.* 23 47–61. 10.4161/cam.25651 9063586

[B95] McComb R. D. Miller K. A. Carson S. D. (1991). Tissue factor antigen in senile plaques of Alzheimer’s disease. *Am. J. Pathol.* 139 491–494. 1887858 PMC1886219

[B96] Mead J. R. Irvine S. A. Ramji D. P. (2002). Lipoprotein lipase: structure, function, regulation, and role in disease. *J. Mol. Med.* 80 753–769. 10.1007/s00109-002-0384-9 12483461

[B97] Meek R. L. Benditt E. P. (1986). Amyloid A gene family expression in different mouse tissues. *J. Exp. Med.* 164 2006–2017. 10.1084/jem.164.6.2006 3783088 PMC2188489

[B98] Mejhert N. Galitzky J. Pettersson A. T. Bambace C. Blomqvist L. Bouloumié A. (2010). Mapping of the fibroblast growth factors in human white adipose tissue. *J. Clin. Endocrinol. Metab.* 95 2451–2457. 10.1210/jc.2009-2049 20228166

[B99] Melchor J. P. Pawlak R. Strickland S. (2003). The tissue plasminogen activator-plasminogen proteolytic cascade accelerates amyloid-β (Aβ) degradation and inhibits Aβ-induced neurodegeneration. *J. Neurosci.* 23 8867–8871. 10.1523/jneurosci.23-26-08867.2003 14523088 PMC6740393

[B100] Miao J. Benomar Y. Al Rifai S. Poizat G. Riffault L. Crépin D. (2018). Resistin inhibits neuronal autophagy through Toll-like receptor 4. *Jo. Endocrinol.* 238 77–89. 10.1530/JOE-18-0096 29773580

[B101] Mohler E. G. Browman K. E. Roderwald V. A. Cronin E. A. Markosyan S. Bitner R. S. (2011). Acute inhibition of 11β-hydroxysteroid dehydrogenase type-1 improves memory in rodent models of cognition. *J. Neurosci.* 31 5406–5413. 10.1523/jneurosci.4046-10.2011 21471376 PMC6622712

[B102] Moreno-Navarrete J. M. Martínez-Barricarte R. Catalán V. Sabater M. Gómez-Ambrosi J. Ortega F. J. (2010). Complement factor H is expressed in adipose tissue in association with insulin resistance. *Diabetes* 59 200–209. 10.2337/db09-0700 19833879 PMC2797922

[B103] Naderali E. K. Ratcliffe S. H. Dale M. C. (2009). Obesity and Alzheimer’s disease: a link between body weight and cognitive function in old age. *Am. J. Alzheimer’s Dis.* 24 445–449. 10.1177/1533317509348208 19801534 PMC10846277

[B104] Nicholson T. E. Renton K. W. (1999). Modulation of cytochrome P450 by inflammation in astrocytes. *Brain Res.* 827 12–18. 10.1016/s0006-8993(99)01261-5 10320688

[B105] Nies V. J. M. Sancar G. Liu W. van Zutphen T. Struik D. Yu R. T. (2016). Fibroblast growth factor signaling in metabolic regulation. *Front. Endocrinol.* 6:193. 10.3389/fendo.2015.00193 26834701 PMC4718082

[B106] Nishitsuji K. Hosono T. Uchimura K. Michikawa M. (2011). Lipoprotein lipase is a novel amyloid beta (Abeta)-binding protein that promotes glycosaminoglycan-dependent cellular uptake of Abeta in astrocytes. *J. Biol. Chem.* 286 6393–6401. 10.1074/jbc.M110.172106 21177248 PMC3057806

[B107] O’Bryant S. E. Johnson L. Edwards M. Soares H. Devous M. D. Ross S. (2013). The link between C-reactive protein and Alzheimer’s disease among Mexican Americans. *J. Alzheimer’s Dis.* 34 701–706. 10.3233/jad-122071 23254637 PMC3608400

[B108] Ouchi N. Kihara S. Funahashi T. Matsuzawa Y. Walsh K. (2003). Obesity, adiponectin and vascular inflammatory disease. *Curr. Opin. Lipidol.* 14 561–566. 10.1097/00041433-200312000-00003 14624132

[B109] Ouchi N. Parker J. L. Lugus J. J. Walsh K. (2011). Adipokines in inflammation and metabolic disease. *Nat. Rev. Immunol.* 11 85–97. 10.1038/nri2921 21252989 PMC3518031

[B110] Oyama R. Yamamoto H. Titani K. (2000). Glutamine synthetase, hemoglobin α-chain, and macrophage migration inhibitory factor binding to amyloid β-protein: their identification in rat brain by a novel affinity chromatography and in Alzheimer’s disease brain by immunoprecipitation. *Biochim. Biophys. Acta* 1479 91–102. 10.1016/s0167-4838(00)00057-1 11004532

[B111] Peng L. Yu Y. Liu J. Li S. He H. Cheng N. (2015). The chemerin receptor CMKLR1 is a functional receptor for amyloid-β peptide. *J. Alzheimer’s Dis.* 43 227–242. 10.3233/jad-141227 25079809

[B112] Pfeiffer A. Schatz H. (1995). Diabetic microvascular complications and growth factors. *Exp. Clin. Endocrinol. Diabetes* 103 7–14. 10.1055/s-0029-1211323 7621107

[B113] Popp J. Bacher M. Kölsch H. Noelker C. Deuster O. Dodel R. (2009). Macrophage migration inhibitory factor in mild cognitive impairment and Alzheimer’s disease. *J. Psychiatr. Res.* 43 749–753. 19038405 10.1016/j.jpsychires.2008.10.006

[B114] Puigoriol-Illamola D. Griñán-Ferré C. Vasilopoulou F. Leiva R. Vázquez S. Pallàs M. (2018). 11β-HSD1 inhibition by RL-118 promotes autophagy and correlates with reduced oxidative stress and inflammation, enhancing cognitive performance in SAMP8 mouse model. *Mol. Neurobiol.* 55 8904–8915. 10.1007/s12035-018-1026-8 29611102

[B115] Radeau T. Lau P. Robb M. McDonnell M. Ailhaud G. McPherson R. (1995). Cholesteryl ester transfer protein (CETP) mRNA abundance in human adipose tissue: relationship to cell size and membrane cholesterol content. *J. Lipid Res.* 36 2552–2561. 8847481

[B116] Radeau T. Robb M. Lau P. Borthwick J. McPherson R. (1998). Relationship of adipose tissue cholesteryl ester transfer protein (CETP) mRNA to plasma concentrations of CETP in man. *Atherosclerosis* 139 369–376. 10.1016/s0021-9150(98)00051-3 9712344

[B117] Reigstad C. S. Lundén G. Ö. Felin J. Bäckhed F. (2009). Regulation of serum amyloid A3 (SAA3) in mouse colonic epithelium and adipose tissue by the intestinal microbiota. *PLoS One* 4:e5842. 10.1371/journal.pone.0005842 19513118 PMC2688757

[B118] Religa P. Cao R. Religa D. Xue Y. Bogdanovic N. Westaway D. (2013). VEGF significantly restores impaired memory behavior in Alzheimer’s mice by improvement of vascular survival. *Sci. Rep.* 3:2053. 10.1038/srep02053 PMC3690383 23792494

[B119] Rodríguez E. Mateo I. Infante J. Llorca J. Berciano J. Combarros O. (2006). Cholesteryl ester transfer protein (CETP) polymorphism modifies the Alzheimer’s disease risk associated with APOE ε4 allele. *J. Neurol.* 253 181–185. 10.1007/s00415-005-0945-2 16096813

[B120] Rosengren A. Skoog I. Gustafson D. Wilhelmsen L. (2005). Body mass index, other cardiovascular risk factors, and hospitalization for dementia. *Arch. Int. Med.* 165 321–326. 15710796 10.1001/archinte.165.3.321

[B121] Rubio-Perez J. M. Morillas-Ruiz J. M. (2012). A review: inflammatory process in Alzheimer’s disease, role of cytokines. *Sci. World J.* 2012:756357. 10.1100/2012/756357 22566778 PMC3330269

[B122] Rupnick M. A. Panigrahy D. Zhang C.-Y. Dallabrida S. M. Lowell B. B. Langer R. (2002). Adipose tissue mass can be regulated through the vasculature. *Proc. Natl. Acad. Sci.* 99 10730–10735. 10.1073/pnas.162349799 12149466 PMC125027

[B123] Sadashiv S. T. Paul B. N. Kumar S. Chandra A. Dhananjai S. Negi M. P. S. (2012). Over expression of resistin in adipose tissue of the obese induces insulin resistance. *World J. Diabetes* 3 135–141. 10.4239/wjd.v3.i7.135 22816026 PMC3399912

[B124] Saijo Y. Kiyota N. Kawasaki Y. Miyazaki Y. Kashimura J. Fukuda M. (2004). Relationship between C-reactive protein and visceral adipose tissue in healthy Japanese subjects. *Diabetes Obes. Metab.* 6 249–258. 10.1111/j.1462-8902.2003.0342.x 15171748

[B125] Samad F. Pandey M. Loskutoff D. J. (1998). Tissue factor gene expression in the adipose tissues of obese mice. *Proc. Natl. Acad. Sci. U.S.A.* 95 7591–7596. 10.1073/pnas.95.13.7591 9636194 PMC22693

[B126] Samanta A. Dawn B. (2016). Il-10 for cardiac autophagy modulation: new direction in the pursuit of perfection. *J. Mol. Cell. Cardiol.* 91 204–206. 10.1016/j.yjmcc.2016.01.002 26772532 PMC4817493

[B127] Sanders A. E. Wang C. Katz M. Derby C. A. Barzilai N. Ozelius L. (2010). Association of a functional polymorphism in the cholesteryl ester transfer protein (CETP) gene with memory decline and incidence of dementia. *JAMA* 303 150–158. 10.1001/jama.2009.1988 20068209 PMC3047443

[B128] Sartipy P. Loskutoff D. J. (2003). Monocyte chemoattractant protein 1 in obesity and insulin resistance. *Proc. Natl. Acad. Sci. U.S.A.* 100 7265–7270. 10.1073/pnas.1133870100 12756299 PMC165864

[B129] Sato S. Kawamura H. Takemoto M. Maezawa Y. Fujimoto M. Shimoyama T. (2009). Halofuginone prevents extracellular matrix deposition in diabetic nephropathy. *Biochem. Biophys. Res. Commun.* 379 411–416. 10.1016/j.bbrc.2008.12.088 19114027

[B130] Scheja L. Heese B. Zitzer H. Michael M. D. Siesky A. M. Pospisil H. (2008). Acute-phase serum amyloid A as a marker of insulin resistance in mice. *Exp. Diabetes Res.* 2008:230837. 10.1155/2008/230837 18584041 PMC2435226

[B131] Schreitmüller B. Leyhe T. Stransky E. Köhler N. Laske C. (2012). Elevated angiopoietin-1 serum levels in patients with Alzheimer’s disease. *Int. J. Alzheimer’s Dis.* 2012 324016. 10.1155/2012/324016 PMC3474986 23094194

[B132] Schulz C. Paulus K. Lehnert H. (2011). “Adipocyte–brain: crosstalk,” in *Sensory and Metabolic Control of Energy Balance*, eds Beisiegel U. Meyerhof W. Beisiegel U., (Berlin: Springer), 189–201. 10.1007/978-3-642-14426-4_16

[B133] Sell H. Laurencikiene J. Taube A. Eckardt K. Cramer A. Horrighs A. (2009). Chemerin is a novel adipocyte-derived factor inducing insulin resistance in primary human skeletal muscle cells. *Diabetes* 58 2731–2740. 10.2337/db09-0277 19720798 PMC2780878

[B134] Sewter C. P. Digby J. E. Blows F. Prins J. O’Rahilly S. (1999). Regulation of tumour necrosis factor-alpha release from human adipose tissue in vitro. *J. Endocrinol.* 163 33–38. 10.1677/joe.0.1630033 10495404

[B135] Shamsi F. Huang T. L. Tseng Y.-H. (2018). Fibroblast growth factor-9 regulates adaptive brown adipose tissue mass expansion. *Am. Diabetes Assoc.* 67:277-OR 10.2337/db18-277-OR

[B136] Shudo K. Fukasawa H. Nakagomi M. Yamagata N. (2009). Towards retinoid therapy for Alzheimer’s disease. *Curr. Alzheimer Res.* 6 302–311. 10.2174/156720509788486581 19519313 PMC2765081

[B137] Skrzypczak-Jankun E. Jankun J. (2010). Theaflavin digallate inactivates plasminogen activator inhibitor: Could tea help in Alzheimer’s disease and obesity? *Int. J. Mol. Med.* 26 45–50. 20514421 10.3892/ijmm_00000433

[B138] Skurk T. Herder C. Kräft I. Müller-Scholze S. Hauner H. Kolb H. (2005). Production and release of macrophage migration inhibitory factor from human adipocytes. *Endocrinology* 146 1006–1011. 10.1210/en.2004-0924 15576462

[B139] Sokolova A. Hill M. D. Rahimi F. Warden L. A. Halliday G. M. Shepherd C. E. (2009). Monocyte chemoattractant protein-1 plays a dominant role in the chronic inflammation observed in Alzheimer’s disease. *Brain Pathol.* 19 392–398. 10.1111/j.1750-3639.2008.00188.x 18637012 PMC8094842

[B140] Sooy K. Noble J. McBride A. Binnie M. Yau J. L. W. Seckl J. R. (2015). Cognitive and disease-modifying effects of 11β-hydroxysteroid dehydrogenase type 1 inhibition in male Tg2576 mice, a model of Alzheimer’s disease. *Endocrinology* 156 4592–4603. 10.1210/en.2015-1395 26305888 PMC4655221

[B141] Sornelli F. Fiore M. Chaldakov G. N. Aloe L. (2009). Adipose tissue-derived nerve growth factor and brain-derived neurotrophic factor: results from experimental stress and diabetes. *Gen. Physiol. Biophys.* 28 179–183. 19893098

[B142] Stefanska A. Sypniewska G. Blaszkiewicz B. Ponikowska I. Cwiklinska-Jurkowska M. (2011). Comparison between C-reactive protein and adipocyte fatty acid-binding protein as a component of metabolic syndrome in middle-aged women. *Clin. Biochem.* 44 304–306. 10.1016/j.clinbiochem.2010.12.002 21159316

[B143] Stopa E. G. Gonzalez A.-M. Chorsky R. Corona R. J. Alvarez J. Bird E. D. (1990). Basic fibroblast growth factor in Alzheimer’s disease. *Biochem. Biophys. Res. Commun.* 171 690–696. 2403357 10.1016/0006-291x(90)91201-3

[B144] Sung H.-K. Doh K.-O. Son J. E. Park J. G. Bae Y. Choi S. (2013). Adipose vascular endothelial growth factor regulates metabolic homeostasis through angiogenesis. *Cell Metab.* 17 61–72. 10.1016/j.cmet.2012.12.010 23312284

[B145] Sutinen E. M. Pirttilä T. Anderson G. Salminen A. Ojala J. O. (2012). Pro-inflammatory interleukin-18 increases Alzheimer’s disease-associated amyloid-β production in human neuron-like cells. *J. Neuroinflam.* 9:199. 10.1186/1742-2094-9-199 PMC3458954 22898493

[B146] Szczepanik A. M. Funes S. Petko W. Ringheim G. E. (2001). IL-4, IL-10 and IL-13 modulate Aβ (1–42)-induced cytokine and chemokine production in primary murine microglia and a human monocyte cell line. *J. Neuroimmunol.* 113 49–62. 10.1016/s0165-5728(00)00404-5 11137576

[B147] Tabata M. Kadomatsu T. Fukuhara S. Miyata K. Ito Y. Endo M. (2009). Angiopoietin-like protein 2 promotes chronic adipose tissue inflammation and obesity-related systemic insulin resistance. *Cell Metab.* 10 178–188. 10.1016/j.cmet.2009.08.003 19723494

[B148] Takahashi M. Suzuki E. Kumano S. Oba S. Sato T. Nishimatsu H. (2013). Angiopoietin-1 mediates adipose tissue-derived stem cell-induced inhibition of neointimal formation in rat femoral artery. *Circ. J.* 77 1574–1584. 10.1253/circj.cj-12-0930 23486192

[B149] Tarkowski E. Liljeroth A.-M. Minthon L. Tarkowski A. Wallin A. Blennow K. (2003). Cerebral pattern of pro-and anti-inflammatory cytokines in dementias. *Brain Res. Bull.* 61 255–260. 10.1016/s0361-9230(03)00088-1 12909295

[B150] Teixeira A. L. Diniz B. S. Campos A. C. Miranda A. S. Rocha N. P. Talib L. L. (2013). Decreased levels of circulating adiponectin in mild cognitive impairment and Alzheimer’s disease. *Neuromol. Med.* 15 115–121. 10.1007/s12017-012-8201-2 23055000

[B151] Thambisetty M. Hye A. Foy C. Daly E. Glover A. Cooper A. (2008). Proteome-based identification of plasma proteins associated with hippocampal metabolism in early Alzheimer’s disease. *J. Neurol.* 255 1712–1720. 10.1007/s00415-008-0006-8 19156487

[B152] Tobinick E. Gross H. Weinberger A. Cohen H. (2006). TNF-alpha modulation for treatment of Alzheimer’s disease: a 6-month pilot study. *Medscape Gen. Med.* 8:25. 16926764 PMC1785182

[B153] Toledo J. B. Korff A. Shaw L. M. Trojanowski J. Q. Zhang J. (2014). Low levels of cerebrospinal fluid complement 3 and factor H predict faster cognitive decline in mild cognitive impairment. *Alzheimer’s Res. Ther.* 6:36. 10.1186/alzrt266 25478014 PMC4255518

[B154] Trayhurn P. Beattie J. H. (2001). Physiological role of adipose tissue: white adipose tissue as an endocrine and secretory organ. *Proc. Nutr. Soc.* 60 329–339. 10.1079/pns200194 11681807

[B155] Tripathy D. Thirumangalakudi L. Grammas P. (2010). RANTES upregulation in the Alzheimer’s disease brain: a possible neuroprotective role. *Neurobiol. Aging* 31 8–16. 10.1016/j.neurobiolaging.2008.03.009 18440671 PMC2803489

[B156] Tsuboi Y. Kakimoto K. Nakajima M. Akatsu H. Yamamoto T. Ogawa K. (2003). Increased hepatocyte growth factor level in cerebrospinal fluid in Alzheimer’s disease. *Acta Neurol. Scand.* 107 81–86. 10.1034/j.1600-0404.2003.02089.x 12580855

[B157] Tsurutani Y. Fujimoto M. Takemoto M. Irisuna H. Koshizaka M. Onishi S. (2011). The roles of transforming growth factor-β and Smad3 signaling in adipocyte differentiation and obesity. *Biochem. Biophys. Res. Commun.* 407 68–73. 10.1016/j.bbrc.2011.02.106 21356196

[B158] Uhlar C. M. Whitehead A. S. (1999). Serum amyloid A, the major vertebrate acute-phase reactant. *Eur. J. Biochem.* 265 501–523. 10.1046/j.1432-1327.1999.00657.x 10504381

[B159] Une K. Takei Y. A. Tomita N. Asamura T. Ohrui T. Furukawa K. (2011). Adiponectin in plasma and cerebrospinal fluid in MCI and Alzheimer’s disease. *Eur. J. Neurol.* 18 1006–1009. 10.1111/j.1468-1331.2010.03194.x 20727007

[B160] Vagnucci A. H. Jr. Li W. W. (2003). Alzheimer’s disease and angiogenesis. *Lancet* 361 605–608. 12598159 10.1016/S0140-6736(03)12521-4

[B161] van Himbergen T. M. Beiser A. S. Ai M. Seshadri S. Otokozawa S. Au R. (2012). Biomarkers for insulin resistance and inflammation and the risk for all-cause dementia and Alzheimer disease: results from the Framingham Heart Study. *Arch. Neurol.* 69 594–600. 22213409 10.1001/archneurol.2011.670 PMC3512190

[B162] Vargas T. Martinez-Garcia A. Antequera D. Vilella E. Clarimon J. Mateo I. (2011). IGF-I gene variability is associated with an increased risk for AD. *Neurobiol. Aging* 32 556–e3. 10.1016/j.neurobiolaging.2010.10.017 21176999

[B163] Wang S. Gu K. (2018). Insulin-like growth factor 1 inhibits autophagy of human colorectal carcinoma drug-resistant cells via the protein kinase B/mammalian target of rapamycin signaling pathway. *Mol. Med. Rep.* 17 2952–2956. 10.3892/mmr.2017.8272 29257307 PMC5783513

[B164] Warren M. W. Hynan L. S. Weiner M. F. (2012). Lipids and adipokines as risk factors for Alzheimer’s disease. *J. Alzheimer’s Dis.* 29 151–157. 10.3233/JAD-2012-111385 22232009 PMC3732377

[B165] Warren R. S. Starnes H. F. Gabrilove J. L. Oettgen H. F. Brennan M. F. (1987). The acute metabolic effects of tumor necrosis factor administration in humans. *Arch. Surg.* 122 1396–1400. 3689116 10.1001/archsurg.1987.01400240042007

[B166] Whitmer R. A. Gunderson E. P. Barrett-Connor E. Quesenberry C. P. Yaffe K. (2005). Obesity in middle age and future risk of dementia: a 27 year longitudinal population based study. *BMJ* 330:1360. 10.1136/bmj.38446.466238.e0 15863436 PMC558283

[B167] Wittamer V. Franssen J.-D. Vulcano M. Mirjolet J.-F. Le Poul E. Migeotte I. (2003). Specific recruitment of antigen-presenting cells by chemerin, a novel processed ligand from human inflammatory fluids. *J. Exp. Med.* 198 977–985. 10.1084/jem.20030382 14530373 PMC2194212

[B168] Wood I. S. Wang B. Jenkins J. R. Trayhurn P. (2005). The pro-inflammatory cytokine IL-18 is expressed in human adipose tissue and strongly upregulated by TNFα in human adipocytes. *Biochem. Biophys. Res. Commun.* 337 422–429. 10.1016/j.bbrc.2005.09.068 16188228

[B169] Wu H. Ghosh S. Perrard X. D. Feng L. Garcia G. E. Perrard J. L. (2007). T-cell accumulation and regulated on activation, normal T cell expressed and secreted upregulation in adipose tissue in obesity. *Circulation* 115 1029–1038. 10.1161/circulationaha.106.638379 17296858

[B170] Xia F. Deng C. Jiang Y. Qu Y. Deng J. Cai Z. (2018). IL4 (interleukin 4) induces autophagy in B cells leading to exacerbated asthma. *Autophagy* 14 450–464. 10.1080/15548627.2017.1421884 29297752 PMC5915013

[B171] Yamada T. Tsubouchi H. Daikuhara Y. Prat M. Comoglio P. M. McGeer P. L. (1994). Immunohistochemistry with antibodies to hepatocyte growth factor and its receptor protein (c-MET) in human brain tissues. *Brain Res.* 637 308–312. 10.1016/0006-8993(94)91250-5 8180811

[B172] Yang Q. Graham T. E. Mody N. Preitner F. Peroni O. D. Zabolotny J. M. (2005). Serum retinol binding protein 4 contributes to insulin resistance in obesity and type 2 diabetes. *Nature* 436 356–362. 10.1038/nature03711 16034410

[B173] Yang R.-Z. Lee M.-J. Hu H. Pollin T. I. Ryan A. S. Nicklas B. J. (2006). Acute-phase serum amyloid A: an inflammatory adipokine and potential link between obesity and its metabolic complications. *PLoS Med.* 3:e287. 10.1371/journal.pmed.0030287 16737350 PMC1472697

[B174] Yao Z. Yang Z. Chen F. Jiang Y. Fu C. Wang Y. (2020). Autophagy is essential for the endothelial differentiation of breast cancer stem-like cells. *Int. J. Mol. Med.* 45 255–264. 10.3892/ijmm.2019.4399 31746369

[B175] Yarchoan M. Louneva N. Xie S. X. Swenson F. J. Hu W. Soares H. (2013). Association of plasma C-reactive protein levels with the diagnosis of Alzheimer’s disease. *J. Neurol. Sci.* 333 9–12. 10.1016/j.jns.2013.05.028 23978419 PMC3815534

[B176] Yu S. Liu Y.-P. Liu H.-L. Li J. Xiang Y. Liu Y.-H. (2018). Serum protein-based profiles as novel biomarkers for the diagnosis of Alzheimer’s disease. *Mol. Neurobiol.* 55 3999–4008. 10.1007/s12035-017-0609-0 28567666

[B177] Yuan H. Li Z.-M. Shao J. Ji W.-X. Xia W. Lu S. (2017). FGF2/FGFR1 regulates autophagy in FGFR1-amplified non-small cell lung cancer cells. *J. Exp. Clin. Cancer Res.* 36:72. 10.1186/s13046-017-0534-0 28558758 PMC5450166

[B178] Zezulak K. M. Green H. (1986). The generation of insulin-like growth factor-1–sensitive cells by growth hormone action. *Science* 233 551–553. 10.1126/science.3726546 3726546

[B179] Zhang R. Reisin E. (2000). *Obesity-Hypertension: The Effects on Cardiovascular and Renal Systems.* Oxford: Oxford University Press. 10.1016/s0895-7061(00)01254-1 11130776

